# A review of redox signaling and the control of MAP kinase pathway in plants

**DOI:** 10.1016/j.redox.2016.12.009

**Published:** 2016-12-09

**Authors:** Yukun Liu, Chengzhong He

**Affiliations:** a Key Laboratory for Forest Resources Conservation and Utilization in the Southwest Mountains of China, Ministry of Education, College of Forestry, Southwest Forestry University, 300 Bailong Si, Kunming 650224, Yunnan, People's Republic of China; b Key Laboratory for Forest Genetic and Tree Improvement & Propagation in Universities of Yunnan Province, College of Forestry, Southwest Forestry University, 300 Bailong Si, Kunming 650224, Yunnan, People's Republic of China

**Keywords:** Abiotic stress, Gene expression, MAPK signaling, Programmed cell death, Plant immunity, Reactive oxygen species

## Abstract

Mitogen-activated protein kinase (MAPK) cascades are evolutionarily conserved modules among eukaryotic species that range from yeast, plants, flies to mammals. In eukaryotic cells, reactive oxygen species (ROS) has both physiological and toxic effects. Both MAPK cascades and ROS signaling are involved in plant response to various biotic and abiotic stresses. It has been observed that not only can ROS induce MAPK activation, but also that disturbing MAPK cascades can modulate ROS production and responses. This review will discuss the potential mechanisms by which ROS may activate and/or regulate MAPK cascades in plants. The role of MAPK cascades and ROS signaling in regulating gene expression, stomatal function, and programmed cell death (PCD) is also discussed. In addition, the relationship between Rboh-dependent ROS production and MAPK activation in PAMP-triggered immunity will be reviewed.

## 1 Introduction

Reactive oxygen species (ROS), including hydrogen peroxide (H_2_O_2_), hydroxyl radical (HO^•^), singlet oxygen (^1^O_2_), and superoxide anion (O_2_^•−^), are formed upon partial reduction of oxygen (O_2_). In plants, ROS are inevitably generated as the by-products of cellular respiration, photosynthesis, protein folding, and a number of metabolic reactions [1]. In mitochondria, O_2_^•−^ is formed by the mitochondrial electron transport chain at the respiratory complexes I, II, and III. O_2_^•−^ can then dismutate to form H_2_O_2_, either spontaneously or catalytically by mitochondrial superoxide dismutase (SOD) [2]. During photosynthesis, various types of ROS are generated by different pathways in chloroplasts [1]. For instance, ^1^O_2_ is produced constitutively in light via chlorophyll [3]. Photoreduction of O_2_ by the reducing side of photosystem II (PSII) produces O_2_^•−^ which dismutates to form H_2_O_2_[4]. On the PSII electron donor side, H_2_O_2_ can be produced by inhibition of enzymatic activity of the water-splitting manganese complex coupled with incomplete oxidation of water. PSI produces O_2_^•−^ by donating an electron to O_2_ instead of ferredoxin [3]. In peroxisomes, H_2_O_2_ is generated as an end product by oxidation pathways such as the glycolate oxidation and β-oxidation of long-chain fatty acids [5]. In the apoplast, O_2_^•−^ can be produced by plasma membrane-located respiratory burst oxidase homolog (Rboh) proteins (also known as plant NADPH oxidases). The O_2_^•−^ dismutates to form H_2_O_2_, spontaneously or catalytically by apoplastic SOD [6]·H_2_O_2_ can also be generated by class III peroxidases in the cell wall, e.g. in response to pathogen attack [7]. Ozone (O_3_) can be degraded into secondary ROS, including H_2_O_2_, O_2_^•−^, and HO^•^ by reacting with components of the cell walls, cellular membranes, and apoplastic fluids [8]. ROS formation occurs under normal growth conditions but can be accelerated when plants are challenged by biotic or abiotic stresses, e.g. plant-pathogen interactions, high light, salinity, and drought [9], [10]. Furthermore, ROS production in these subcellular compartments is not isolated [11]. ROS generation in specific subcellular compartments may lead to ROS accumulation in other compartments.

High levels of ROS can lead to detrimental effects (ROS stress). ROS stress causes direct or indirect ROS-mediated damage of a variety of molecules including lipid peroxidation in cellular membranes, protein denaturation, carbohydrate oxidation, and pigment breakdown [12]. ROS stress can eventually lead to changes in gene expression and even cell death. However, ROS stress can be controlled by ROS-scavenging systems. Generally, the ROS-scavenging systems are divided into enzymatic and nonenzymatic mechanisms [1]. Enzymatic ROS-scavenging mechanisms include a battery of antioxidant enzymes such as superoxide dismutase (SOD), catalase (CAT), peroxidase (PRX), ascorbate peroxidase (APX), glutathione peroxidase (GPX), and dehydroascorbate reductase (DHAR) [1]. Antioxidant enzymes exist in multiple isoforms. For instance, SODs are classified into three groups based on the metal cofactors: iron SOD (Fe-SOD) is predominantly localized in the chloroplast, manganese SOD (Mn-SOD) is localized in the mitochondria and peroxisomes, and copper/zinc SOD (Cu/Zn-SOD) is localized in the cytosol and chloroplast [13], [14], [15]. Non-enzymatic ROS scavenging mechanisms consist of soluble molecules which can be oxidized by ROS. Glutathione (GSH) and ascorbate (ASC) are the major non-enzymatic systems by which H_2_O_2_ can be reduced to H_2_O and O_2_ [16]. Maintenance of the reduced and oxidized glutathione ratio (GSH/GSSG) is critical for ROS homeostasis.

In contrast to their potentially harmful effects, ROS play an important role in regulating plant growth and development, and response to biotic or abiotic stresses. It has long been observed that a virulent pathogens, successfully recognized by plants via R proteins, induce a biphasic ROS production with a low-amplitude and transient first phase (ROS burst) followed by a sustained phase of much higher magnitude [17], [18]. In abiotic stress, the biphasic increases of ROS production can also be observed, for instance, during ozone or wounding stress [18], [19]. It has been demonstrated that, in particular tissues of *Arabidopsis thaliana*, cold, heat, high light, wounding, or salinity triggers AtRbohD-dependent ROS production. This local production of ROS can initiate rapid and long-distance signals traveled from the affected tissue to the entire plant, which has been shown to be accompanied by apoplastic ROS production and depends on AtRbohD [20]. Further research indicates a model that ROS induced ROS release (termed the ROS wave [21]), coupled with calcium signaling and other systemic signals (e.g. abscisic acid (ABA), hydraulic waves, and electric signals), plays an important role in abiotic stresses [22], [23], [24]. Furthermore, in response to *Pseudomonas syringae* pathovar *tomato* (*Pst*) DC3000, it was demonstrated that AtRbohD could also facilitate a long-distance signal for activation of defense response at distal sites [25].

The mitogen-activated protein kinase (MAPK or MPK) cascade is an intracellular pathway conserved among eukaryotes. A MAPK cascade is composed of three kinases, MAPK, MAPK kinase (MKK), and MAPKK kinases (MAPKKK or MEKK) [26]. The three kinases form the basic module of a MAPK cascade, MAPKKK-MKK-MAPK, in which MAPKKK phosphorylates and activates MKK, and the activated MKK phosphorylates and activates MAPK [27]. MAPK cascades are involved in a many aspects of plant physiology, including cell division [28], plant growth and development [29], plant resistance to pathogens [30] and insect herbivores [31], and plant response to abiotic stresses [32], [33].

It has been observed that, on one hand, exogenous application of H_2_O_2_ or ozone activates components of MAPK cascades, and initiation of ROS signaling can lead to changes in MAPK cascades (Table 1; [34], [35]), on the other hand, manipulating MAPK cascades results in initiation of ROS responses (Table 2; [34], [35]). These observations indicate that MAPK cascades are complicated in both ROS signaling and responses.

## 2 Activation and regulation of MAPK cascades by ROS

Experimental results indicate that O_2_ deprivation leads to mitochondria-dependent ROS production in *A. thaliana* [36]. Under O_2_ deprivation, as well as application of cell respiration inhibitors antimycin A (AA) or potassium cyanide (KCN), resulted in ROS production and transient activation of AtMPK6 and AtMPK3 [36]. The authors suggested that low O_2_ stimulates mitochondrial ROS that moves to the cytoplasm to activate MAPKs, leading to retrograde signaling between mitochondria and nucleus. As the authors point out, however, the O_2_ deprivation treatment also removes carbon dioxide. In addition, the inhibitors AA and KCN may affect photosynthesis. Therefore, it cannot be excluded that, in green leaves, signaling from the chloroplast may also participate in activating AtMPK6 and AtMPK3. Recently, it has been shown that, when *A. thaliana* plants shifted from low-light to high-light 8–800 μmol quanta m^−2^ s^−^^1^), AtMPK6 can be activated in retrograde signaling between chloroplast and nucleus. In this signaling, AtMPK6 regulates gene expression of several key transcriptional factors, e.g. *ERF6* and *ERF104*, possibly prior to H_2_O_2_ accumulation [37].

Exogenous application of H_2_O_2_ has been shown to activate components of MAPK cascade (Table 1). In *A. thaliana*, AtMPK6 and AtMPK3 can be activated by various concentration of H_2_O_2_. Experiments in protoplasts showed that activation of AtMPK6 and AtMPK3 requires ANP1 (a MAPKKK) that itself can be activated by H_2_O_2_ [38], and AtMKK4-AtMPK6 and AtMKK3-AtMPK7 may be two pathways involved in H_2_O_2_ responses [39]. H_2_O_2_ also activates an oxidative signal inducible1 (OXI1) kinase that is required for full activation of AtMPK6 and AtMPK3 [40]. Exposure of acute ozone (high dose within a short time frame, e.g. 250–700 nl L^–1^ within 24 h) activates AtMPK3/AtMPK6 in *A. thaliana* [41] and SIPK/WIPK in tobacco [42], which has been shown to be important for ozone sensitivity.

**Fig. 1:**
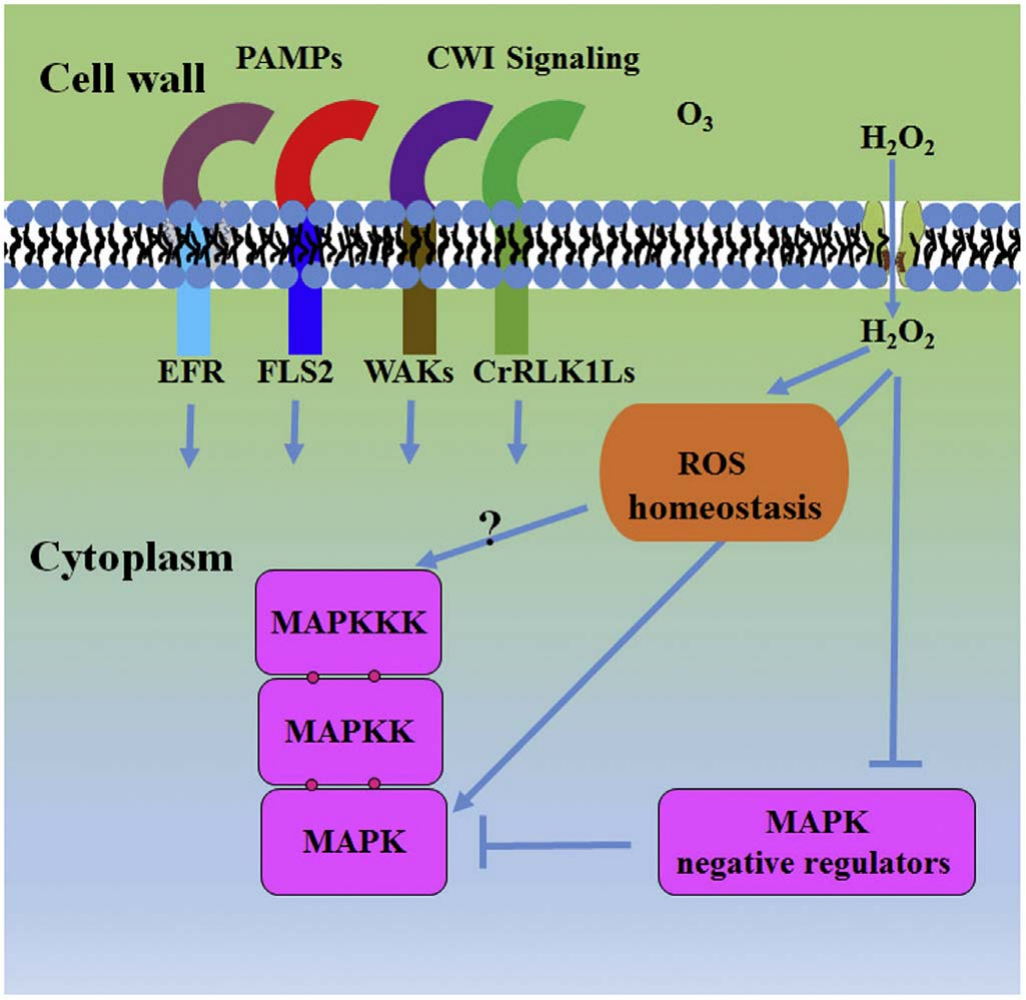
**Putative mechanisms for activation and regulation of MAPK cascade by ROS**. ROS are generated both intracellularly and apoplastically, and rapidly removed by antioxidant proteins (ROS homeostasis). Once ROS production exceeds the capacity of the antioxidant proteins, ROS may induce oxidative modification of MAPK signaling proteins, thereby leading to MAPK activation. ROS may also activate MAPK signaling via inhibition and/or degradation of MAPK negative regulators, e.g. PTPs or MKPs. ROS may directly act on MAPK protein, e.g. by H_2_O_2_-dependent sulfenylation. Extracellular ROS may change the activity of receptor-like kinases by redox dependent modifications to activate MAPK signaling, e.g. in cell wall metabolism and development.

One way that changes in apoplastic redox status trigger intracellular signaling is likely by H_2_O_2_·H_2_O_2_ has the longest lifetime among the various ROS and can be transported across the plasma membrane [43]. Although H_2_O_2_ could simply diffuse across the plasma membrane, it is suggested that H_2_O_2_ may preferentially enter the cell by aquaporin channels [44], [45], [46]. This regulated entry may provide another way of adjusting local concentrations of H_2_O_2_. If H_2_O_2_ can be channeled to an intended intracellular compartment, it is speculated that this could provide a layer of specificity in ROS signaling. Inside the cell, while it is possible that H_2_O_2_ may initiate MAPK cascades directly, it is indicated that the prominent role of H_2_O_2_ is to disturb ROS homeostasis via a redox relay mechanism [47]. As a result of these disturbances, intracellular levels of ROS may rapidly rise, transferring the oxidation to target MAPK cascades. Indeed, this mechanism has been demonstrated in mammalian cells·H_2_O_2_-induced oxidation of thioredoxin (Trx) leads to the conformational change of Trx and disruption of interaction between Trx and apoptosis signal-regulating kinase 1 (ASK1), resulting in ASK1 activation and subsequent phosphorylation of its substrate p38 MAPK [48]. In plants, activation of MAPKKKs, e.g. ANP1, OMTK, or MEKK1, by H_2_O_2_ has been observed (Table 1). It remains to be seen if a redox sensor could activate the MAPKKK in a redox-dependent manner. In *Nicotiana tabacum* (tobacco), recent study showed that infiltration of either reduced glutathione (GSH) or the oxidized form (GSSG) could activate SIPK and WIPK [49]. Transgenic tobacco expressing the bifunctional glutathione biosynthetic enzyme from *Streptococcus thermophilus* (StGCL-GS) showed increased levels of glutathione accumulation and displayed activation of SIPK and to a lesser extent of WIPK [49]. These suggest that the oxidative shift in cytosolic redox potential could activate MAPK signaling (Fig. 1).

H_2_O_2_ may also activate MAPK by inactivating the MAPK repressors. *Arabidopsis* genome encodes five dual-specificity MAPK phosphatases (MKPs), dual-specificity protein tyrosine phosphatase 1 (AtDsPTP1), MAP kinase phosphatase 2 (AtMKP2), indole-3-butyric acid response 5 (IBR5), propyzamide hypersensitive 1 (PHS1), and MAP kinase phosphatase 1 (AtMKP1) [50], [51]. Among these, DsPTP1 [52], AtMKP1 [53], [54], and AtMKP2 [55] have been implicated in ROS signaling and response. In the Wassilewskija (WS) ecotype *A. thaliana*, *atmkp1* mutants produce higher ROS in response to elf18 (synthetic polypeptide that corresponds to the bacterial PAMP elongation factor-Tu, EF-Tu; [53]). *AtMKP2*-RNAi plants are hypersensitive to ozone treatment and display significantly prolonged AtMPK3 and AtMPK6 activation [55]. Over-accumulation of H_2_O_2_ or O_2_^•−^ was observed in response to methyl viologen (MV) in *atmkp2* mutants [56]. Furthermore, it has been shown that both AtDsPTP1 and AtPTP1 (one of the Tyr phosphatases) could dephosphorylate and inactivate MAPKs [52], [57]. The activity of AtPTPs and AtDsPTPs requires a highly conserved Cys residue: Cys265 in AtPTP1 [57] and Cys135 in AtDsPTP1 [52]. Particularly, the inactivation of AtMPK6 by AtPTP1 *in vitro* and *in vivo* is negatively influenced by H_2_O_2_ treatment, suggesting that AtPTP1 may act as a redox sensor and H_2_O_2_ may activate AtMPK6 by inhibiting AtPTP1 [58] (Fig. 1).

There are also experiments that indicate a mechanism that ROS may directly act on the MAPK protein. A recent study of proteins with cysteine thiol oxidized to a sulfenic acid showed that AtMPK2, AtMPK4, and AtMPK7 undergo H_2_O_2_-dependent sulfenylation [59]. Indeed, it has been shown that AtMPK2, AtMPK4, and AtMPK7 can be activated by H_2_O_2_ (Table 1; [39], [60], [61]. However, the biological significance of H_2_O_2_-dependent MAPK sulfenylation remains to be determined. Since exogenous application of H_2_O_2_ cannot induce the activation of AtMPK4 in *mekk1* mutants [62], [63], it seems Cys oxidation of AtMPK4 cannot lead to its activation. Recently, the BnMPK4, the orthologue of AtMPK4 in *Brassica napus*, has been shown to be activated by H_2_O_2_ and to undergo H_2_O_2_-dependent aggregation. The aggregation of BnMPK4 was abolished by mutation of Cys232 [64]. Therefore, it is possible that MAPKs can be modified by Cys oxidation, resulting in changes to their aggregation, stability, or interaction with other proteins; but activation solely depends on phosphorylation by upstream MKK.

In addition, apoplastic ROS are involved in cell wall integrity (CWI) signaling [65]. Potential CWI sensors include the members of wall-associated kinases (WAKs) and *Catharanthus roseus* RLK1-like kinases (CrRLK1Ls). It is possible that apoplastic ROS may activate MAPK signaling by CWI sensors, e.g. in cell wall metabolism and development [65], [66]. Apoplastic ROS may also directly change the activity of extracellular proteins by redox-dependent modifications [67]. In response to ozone, it is hypothesized that cysteine-rich receptor-like kinases (CRKs) are good candidates for relaying extracellular ROS signaling to intracellular MAPK activation [8] (Fig. 1).

## 3 Regulation of ROS-related genes by MAPK cascades

**Fig. 2:**
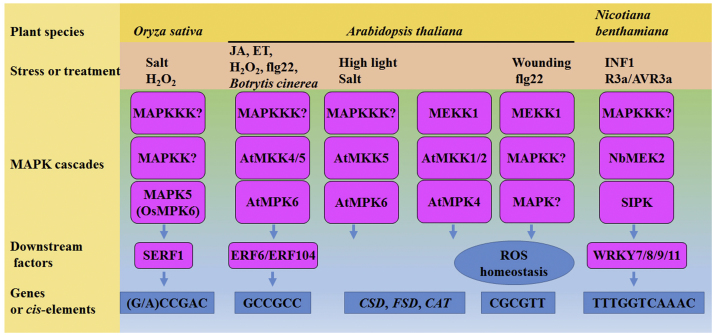
**Putative regulation of ROS-related genes by MAPK cascades**. Under salt stress, rice MAPK5 (OsMPK6) regulates H_2_O_2_-responsive genes by DREB-specific *cis*-element GCCGAC or ACCGAC. ERF6 and ERF104 are two substrates of AtMPK6 and can regulate stress-responsive genes via GCC box (GCCGCC). AtMPK6-ERF6 pathway may regulate ROS-responsive gene via GCC box. Gene expression of ROS scavenging enzymes are changed in *atmpk6* (under high light or salt stress) or *atmpk4* mutants. Defective MEKK1-AtMKK1/AtMKK2-AtMPK4 cascade affects ROS homeostasis. MEKK1 pathway may regulate stress-responsive genes possessing *cis*-element CGCGTT in wounding by ROS signaling. In *N. benthamiana*, *Nb*MKK2-mediated MAPK signaling regulate *cis*-element (TTTGGTCAAAC) of the *NbRbohB* promoter by phosphorylating WRKYs (e.g. WRKY7/WRKY8/WRKY9/WRKY11) in response to INF1 or R3a/AVR3a.

In *A. thaliana*, MEKK1–AtMKK1/AtMKK2–AtMPK4 cascade is involved in ROS homeostasis. Transcriptional analysis showed that some redox-related genes are misexpressed in *mekk1* or *atmpk4* mutant plants [62], [63]. The microarray analysis concerning MEKK1–AtMKK1/AtMKK2–AtMPK4 cascade revealed that, on one hand, there is a great deal of overlap among ROS-related genes in *mekk1*, *atmkk1*/*atmkk2*, and *atmpk4* mutants. On the other hand, some ROS-related genes, e.g. *CSD1* (encoding Cu/Zn-SOD1), *CAT1*, and *APX1*, are differentially expressed between *mekk1*, *atmkk1*/*atmkk2*, and *atmpk4* mutants [68]. Among the genes encoding ROS scavenging enzymes that are crucial for redox homeostasis, *CAT2* expression is down-regulated in *mekk1* and *atmpk4* mutants, but unaffected in *atmkk1*/*atmkk2* double mutants. Furthermore, the steady-state transcripts of *CAT1* and *CAT3* are up-regulated in *mekk1* mutants and in *atmkk1*/*atmkk2* double mutants, but not in *atmpk4* mutants [68]. These results suggest that, in regulating ROS scavenging genes, MEKK1, AtMPK4, or AtMKK1/AtMKK2 may function independently of the MEKK1–AtMKK1/AtMKK2–AtMPK4 cascade in response to specific ROS signaling or in different cell-compartments. Furthermore, in *atmpk4* mutants or *atmpk4*/*ics1* double mutants, it was shown that most of the analyzed genes encoding ROS scavenging enzymes, e.g. *GR2* (encoding glutathione reductase 2), *FSDs* (encoding Fe-SODs), and *APXs*, are down-regulated, whereas *CSD2* can be up-regulated [69]. The enzyme analysis demonstrated Cu/Zn-SOD and APX activities were increased whereas Fe-SOD activity was decreased in *atmpk4* mutants or *atmpk4*/*ics1* double mutants [69] (Fig. 2).

Previous studies indicated taht AtMKK1 induce *CAT1* expression by triggering H_2_O_2_ production in response to ABA, drought, and salt stress [70]. Further analysis showed that AtMKK1-AtMPK6 could regulate *CAT1* expression in ABA-induced H_2_O_2_ production [71]. Recently, Xing et al. suggested that AtMKK5-AtMPK6 regulate genes encoding Cu/Zn-SOD (*CSD1* and *CSD2*) in high light ((800 mmolm-2 s^−1^; [14]) and genes encoding Fe-SODs (*FSD2* and *FSD3*) in salt stress [15]. It is likely that the MAPK-regulated gene expression of ROS scavenging enzymes can be regulated by stress-induced ROS production. This regulation may be crucial for redox homeostasis and stress tolerance (Fig. 2).

Studies focusing on general stress response (GSR) in *A. thaliana* revealed that GSR could modulate stress-responsive genes partly by the *cis*-regulatory element CGCGTT known as rapid stress response element (RSRE; [72]). In *mekk1-5* mutants, wounding induced an earlier peak time of RSRE monitored by luciferase *in vivo* when compared with the parent plants, suggesting that, genetically, MEKK1 inhibits the rapid expression of stress-responsive genes possessing CGCGTT in wounding [73]. Pharmacologically, PD98059 (a broad-spectrum inhibitor of MKK) can inhibit the activity peak and can also delay the peak time of RSRE in response to flg22 (a 22-amino acid synthetic polypeptide that corresponds to a highly conserved epitope of the *Pseudomonas* PAMP flagellin), indicating MAPK cascades may regulate the rapid transcription of genes possessing CGCGTT regulatory elements during plant immune responses [73]. The reports also support the idea that RSRE activation is likely regulated by ROS, as (1) the earlier peak time of RSRE is consistent with the over-accumulation of ROS in *mekk1-5* mutants; and (2) MV-induced ^1^O_2_ could activate RSRE [73].

In *A. thaliana*, several transcription factors have been identified that act downstream of MAPKs in ROS responses. These include the MYB-transcription factor 44 (MYB44; [74], [75]), the heat shock transcription factor A4a (HSFA4A; [76]), and the ethylene response factor 6 (ERF6; [77], [78]). Particularly, AtMPK6-ERF6 have been demonstrated to regulate ROS-responsive gene via GCC box (AGCCGCC) in response to biotic and abiotic stresses [77], [78]. Interestingly, AtMPK6-ERF6 can also bind the GCC box to regulate jasmonic acid/ethylene (JA/ET)-responsive genes in response to necrotrophic fungal pathogen *Botrytis cinerea* [79], [80], [81], [82]. It is unclear whether JA/ET and ROS signaling can partly become merged in regulating genes with GCC box in the promoters, e.g. in response to *B. cinerea*. In rice, salt-responsive ERF1 (SERF1) is a transcription factor. Expression of *SERF1* gene can be induced by salt and H_2_O_2_ treatment. Analysis of *SERF1* loss-of-function plants showed that *SERF1*-dependent genes are H_2_O_2_ responsive. Further analysis indicated that SERF1 is a substrate of MAPK5 (OsMPK6). SERF1 regulates H_2_O_2_-responsive genes, e.g. *MAP3K6*, *MAPK5*, *DREB2A*, *ZFP179*, and *SERF1*, by DREB-specific *cis*-element GCCGAC or ACCGAC [83] (Fig. 2).

Besides regulating H_2_O_2_-responsive genes, MAPK signaling has also been implicated in regulating genes encoding Rboh in ROS production. In wounding response, AtMPK8 negatively regulates *AtRbohD* expression. In *atmpk8* mutants, *AtRbohD* expression was higher compared with wild type. Further analysis showed that AtMPK8 negatively regulates ROS accumulation via repressing *AtRbohD* expression [84]. In response to flg22, *AtRbohD* is up-regulated in ERF104-overexpressing plants and ERF104 has been identified to be a substrate of AtMPK6 [85]. In *erf6* mutants, it seems that *AtRbohD* expression can be up-regulated [77]. However, although AtMPK6-ERF6 and AtMPK6-ERF104 have been demonstrated to regulate stress-responsive genes via GCC box, it is unlikely that they directly regulate *AtRbohD* expression via GCC box, since no GCC box was found in the upstream 2500 bp region of *AtRbohD* promoter. The precise role of MAPK signaling in regulating *AtRbohD* expression needs to be elucidated in future studies. In *N. benthamiana*, *NbRbohB* gene, the orthologue of Arabidopsis *AtRbohD*, was shown to be important for ROS production in immunity. It has been shown that *NbRbohB* expression was induced by MAPK signaling (possibly by NbMKK2-SIPK and NbMKK1-NTF6 cascades) in response to INF1, the elicitor produced by the oomycete *Phytophthora* species [86], [87]. The recent discovery demonstrated that *Nb*MKK2-mediated MAPK signaling regulates *cis*-element (TTTGGTCAAAC) of the *NbRbohB* promoter by phosphorylating WRKYs (e.g. WRKY7/WRKY8/WRKY9/WRKY11) in response to INF1 or R3a/AVR3a (the potato R protein/oomycete effector pair) but not flg22 [88] (Fig. 2). In addition, Maize *MAPK5* (*ZmMPK6-2*) is one of the orthologues of Arabidopsis *AtMPK6* [89], [90]. Based on pharmacological evidence, it is proposed that the expression of *ZmRboh* genes could be regulated by *MAPK5* in maize leaves in response to ABA, H_2_O_2_, and brassinosteroids [91], [92].

The mechanisms by which MAPK signaling controls gene expression remain to be elucidated. Experimental data showed that components of MAPK cascade can be detected in the nucleus [93]. AtMPK4 [94], AtMPK6 [85], and SIPK [95] have been implicated in ROS signaling and responses. In addition, in the early stages of O_3_ treatment, it is indicated that activated AtMPK3 and AtMPK6 are translocated to the nucleus where they are thought to phosphorylate substrates and regulate gene expression [41]. Furthermore, AtMPK3/AtMPK6 can phosphorylate ERF6. The stabilization and accumulation of ERF6 were reported to initiate transcriptions of stress genes [82]. It is possible that the mechanisms underlying ROS-related gene expression by MAPK cascades might be different in response to different types of stimuli or depending on the physiological context. ROS may directly regulate transcription factors, e.g·H_2_O_2_ can regulate transcription factors with redox-sensitive Cys residue [96]. The regulation of transcription factors by ROS may influence the conformational changes, nucleocytoplasmic shuttling, proteolysis, or interaction with other regulators [96]. Although it has not been demonstrated, it is possible that some MAPK substrates may be regulated by ROS directly. In this scenario, ROS may lead to simultaneous multisite modifications of the same pathway, which may guarantee efficient or specificity of signaling transduction.

## 4 Induction of cell death by ROS and MAPK cascades

### 4.1 ROS and MAPK cascades in HR-like cell death

The incompatible plant–pathogen interaction can induce plant programmed cell death (PCD) at the infection sites known as hypersensitive response (HR; [97]). HR is often characterized by discrete cellular lesions and preceded by an ROS burst. HR and the accompanying ROS is thought to act against hemi-biotrophic and biotrophic pathogens that obtain their nutrition from living host cells. Initiation of HR may further lead to activation of systemic acquired resistance to avoid the death of the entire plant.

Previous studies revealed that transgenic *A. thaliana* with inducible AtMKK4^DD^ (the constitutively active mutants of AtMKK4 protein) or AtMKK5^DD^ exhibit cell death when AtMKK4^DD^ or AtMKK5^DD^ are induced. The cell death is similar when an HR is induced by infection by the bacterial pathogen *Pst* DC3000 [98]. The HR-like cell death is preceded by H_2_O_2_ generation and AtMPK3/AtMPK6 activation. In tobacco leaves, transient expression and induction of AtMKK4^DD^, AtMKK5^DD^, or NtMKK2^DD^ also leads to PCD with prolonged activation of SIPK/WIPK, the orthologues of AtMPK6/AtMPK3. There are also reports that in *N. tabacum* and *N. benthamiana*, SIPK-mediated MAPK cascade regulate harpin-induced cell death [99] and INF1-induced cell death [100]. Furthermore, Zhang et al., 2012a showed that many bacterial, fungal, and oomycete species secrete necrosis and ethylene-inducing peptide 1 (*Nep1*)-like proteins (NLP) that trigger PCD. Interestingly, the MAPKKKα-NbMKK2-WIPK cascade might regulate Nep1Mo-activated H_2_O_2_ accumulation and contribute to Nep1Mo-triggered cell death, as virus-induced gene silencing WIPK but not SIPK affects Nep1Mo-induced H_2_O_2_ accumulation [101].

The relationship between ROS and AtMPK6/AtMPK3 (SIPK/WIPK) remains elusive. It is suggested that harpin-induced ROS accumulation is required for activation of SIPK and WIPK [99]. In another study, however, it was shown that activation of the SIPK/Ntf4/WIPK cascade by pathogens *Phytophthora cryptogea* promotes the ROS generation in chloroplasts, which plays an important role in executing HR-like cell death [102]. Further studies indicated a rapid inhibition of photosynthetic CO_2_ fixation after NtMKK2^DD^-induced SIPK/Ntf4/WIPK activation, leading to the generation of ROS in chloroplasts under illumination [103]. In the dark, cell death is only delayed but not blocked [103]. Therefore, it seems that the chloroplast is not the sole organelle to execute HR-like cell death. It has been shown that SIPK/WIPK function downstream of HSP90 to transduce the cell death signal to mitochondria in TMV resistance gene N-mediated HR-like cell death [104].

### 4.2 ROS and MAPK cascades in spontaneous cell death

Genetically, MEKK1-AtMKK1/AtMKK2-AtMPK4 cascade has been demonstrated to negatively regulate H_2_O_2_ accumulation and spontaneous cell death [62], [63], [105]. A nucleotide binding leucine-rich repeat (NB-LRR) protein, suppressor of mkk1 mkk2 2 (SUMM2), acts as genetic repressor of MEKK1-AtMKK1/AtMKK2-AtMPK4 cascade [106]. It is assumed that another repressor of MEKK1-AtMKK1/AtMKK2-AtMPK4 cascade, SUMM1 (encoding MEKK2), triggers SUMM2-mediated responses, although it is unclear whether SUMM1 could initiate a MAPK cascade [107]. Therefore, H_2_O_2_ accumulation and spontaneous cell death in a defective MEKK1-AtMKK1/AtMKK2-AtMPK4 cascade may result from the disinhibition of SUMM1 and SUMM2. Shih-Heng Su at al 2013 further showed that upregulation of *MEKK2* RNA abundance is required for the response of defect in MEKK1-AtMKK1/AtMKK2-AtMPK4 cascade [108]. Recently, two substrates, protein associated with topoisomerase II 1 (PAT1; [109]) and Arabidopsis SH4-related 3 (ASR3; [110]), were demonstrated to act downstream of AtMPK4 to negatively regulate gene expression in response to flg22. However, linking AtMPK4 to *MEKK2* expression in spontaneous cell death remains elusive.

L-*myo*-inositol 1-phosphate synthase 1 (MIPS1) is the rate-limiting enzyme in *myo*-inositol biosynthesis. Mutation in *MIPS1* results in spontaneous cell death [111], [112]. It is possible that MIPS1 controls its own transcription by the AtMPK4 pathway, as *MIPS1* expression is down-regulated in *atmpk4* mutant [113]. ROS have been involved in the spontaneous cell death in *mips1* mutants. For instance, *mips1* mutants show increased sensitivity to ROS stress and reduced ascorbic acid levels [112], and a decrease in *myo*-inositol accumulation is necessary for the spontaneous cell death in catalase-deficient *cat2* mutants [114], [115]. Furthermore, the cleavage and polyadenylation specificity factor 30 (CPSF30), encoding a polyadenylation factor subunit homolog, is required for spontaneous cell death in *mips1* or *atmpk4* mutants [116]. It was shown that CPSF30 is a general controller of the salicylic acid (SA) pathway rather than a direct regulator of ROS production or scavenging in the spontaneous cell death [116]. However, in the process of spontaneous cell death, CPSF30 may be connected with ROS signaling and responses by unknown mechanisms, as CPSF30 mutant is more tolerant than wild type in ROS stress [117].

### 4.3 ROS and MAPK cascades in acute ozone-induced PCD

Exposure to acute ozone can induce ROS burst leading to cell death that shares similarities with the HR [19], [118], [119]. Acute ozone induces MAPK cascades (Table 1). Genetic manipulation of MAPK cascades affects the cell death phenotype observed in O_3_ responses (Table 2). Mutations of *radical-induced cell death1* (*rcd1*) show more ozone-sensitive and prolonged activation of AtMPK3/AtMPK6 compared with the wild-type plants [120], indicating that prolonged activation of MAPK signaling may participate in the induction of cell death process in response to acute ozone.

It is possible that regulation of Rboh-dependent ROS production is the initial step in ozone signaling. It was reported that, in *A. thaliana*, exposure of acute ozone induced biphasic ROS generation that is associated with the pathogen response [19]. Recent reports showed that in the JA receptor mutant *coi1-16*, ozone-induced cell death was suppressed by impairment of AtRbohF [121]. This observation is reminiscent of reports showing that AtRbohD/AtRbohF-dependent ROS can suppress SA-dependent HR in cells around infection sites in response to *Pst* DC3000, with AtRbohD having a prominent role [122].

### 4.4 ROS and MAPK cascades in fumonisin B1- and sphingolipid-induced PCD

Fumonisin B1 (FB1) is a specific inhibitor of ceramide synthase. FB1 application can generate an accumulation of LCBs and it has been used to investigate the functions of sphingolipid metabolism in plants [123], [124]. FB1-induced cell death in *A. thaliana* is associated with ROS production, callose deposition, and PR gene expression, which is similar to HR [125]. The mutant *fumonisin B1 resistant11-1* (*fbr11-1*) is defective in a long-chain base1 subunit of serine palmitoyltransferase and is compromised in ROS generation and subsequent induction of PCD when challenged by FB1 [123]. Direct feeding experiments showed that dihydrosphingosine (d18:0), phytosphingosine (t18:0), and sphingosine (d18:1) can induce ROS generation followed by cell death, whereas the phosphorylated form of d18:0 (d18:0-P, dihydrosphingosine-1-phosphate) could block the ROS generation and PCD, indicating that homeostasis of free LCB and its phosphorylated form may determine the cell fate [123]. AtMPK6 plays a regulatory role in PCD induced by LCBs and FB1 [124]. In the *atmpk6* seedling, exogenous application of d18:0 did not lead to severe PCD. The PCD phenotype result from the increase of endogenous LCBs induced by FB1 was attenuated in *atmpk6* seedlings [124]. In another study, it is reported that t18:0-P rapidly and transiently formed in *A. thaliana* upon chilling (4 °C). The activity of AtMPK6 (0–30 min) was stimulated by exogenous t18:0-P [126]. The authors showed that d18:0-P or t18:0 cannot induce AtMPK6 activity at 22 °C or 4 °C, in contrast with the observations that unphosphorylated LCB t18:0 can trigger AtMPK6 activation [124], possibly reflecting differences in the biological material used and in the treatment procedure. It is speculated that AtMPK6 acts downstream of homeostasis between free LCBs and the phosphorylated derivatives. Activation of AtMPK6 may lead to response to chilling stress in the presence of elevated t18:0-P. However, the prolonged activation of AtMPK6 may promote ROS accumulation and PCD in the presence of elevated d18:0.

### 4.5 ROS and MAPK cascades in self-incompatibilily-induced PCD

Self-incompatibility (SI) is a genetic mechanism used by many angiosperm species to prevent inbreeding. Studies in *Papaver rhoeas* showed that recognition of incompatible pollen can trigger signaling to initiate PCD in pollen tubes. This signaling includes activation of a 56 kDa MAPK [127], [128]. The activation of 56 kDa MAPK was shown at 5 min, peaking at 10 min, and lasting up to 30 min after SI induction [127]. Because around 10 min after SI induction may play a pivotal role in determining irreversible PCD of incompatible pollen [127], [128], [129], it seems that a MAPK cascade is involved in initiating SI-induced PCD. Increases in ROS, however, can be detected at 2 min and can last up to 15 min, indicating that ROS signaling acts upstream of the 56 kDa MAPK activation [128], [129]. The possible role of the 56 kDa MAPK cascade is to stimulate nitric oxide (NO) signaling to activate caspase-3-like activity, as increases in NO occurred at 15–40 min after SI induction and pharmacological application of U0126 (the specific MKK inhibitor) can prevent activation of caspase-3-like activity [128], [129].

## 5 Rboh-dependent ROS production and MAPK cascades in PAMP-triggered immunity

Plants use two layers of immune systems to defend against pathogens. The first layer is based on perception of pathogen-associated molecular patterns (PAMPs) by pattern recognition receptors (PRRs) in the cell surface, leading to the PAMP triggered immunity (PTI). The second layer is based on recognition of microbial effectors secreted in cells by resistance (R) proteins, leading to the effector triggered immunity (ETI; [130]). In *A. thaliana*, perception of bacterial PAMPs flagellin and EF-Tu by the corresponding PRRs, flagellin-sensing 2 (FLS2) and EF-Tu receptor (EFR), activates early common responses such as ROS production and MAPK activation ([131] and references therein). In response to flg22 or *Pst* DC3000, the rapid and transient ROS production (about 0–30 min) is dependent on AtRbohD [132], [133], [134], [135], one of the ten Rboh proteins in *A. thaliana* [9]. Quantitative phosphoproteomic analysis showed that phosphorylation of AtRbohD plays an important role in AtRbohD-dependent ROS production [132], [136]. Recent reports demonstrated that the membrane-anchored *Botrytis*-induced kinase 1 (BIK1), a multifunctional receptor-like cytoplasmic kinase, directly regulates AtRbohD-dependent ROS production in response to flg22 or elf18 [137], [138].

**Fig. 3:**
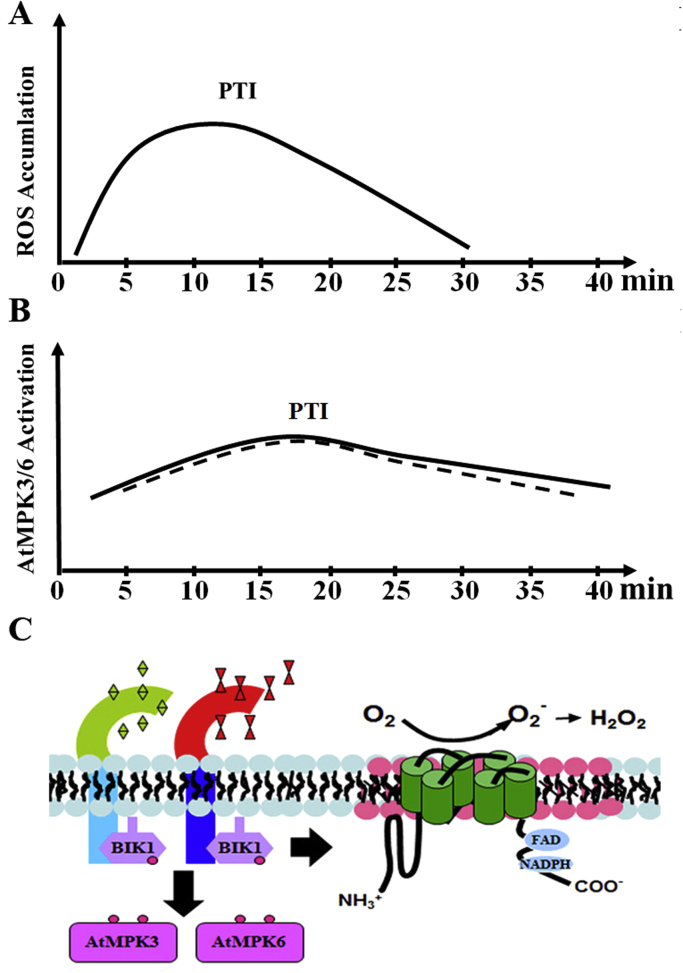
**RbohD-dependent ROS production and AtMPK3/6 activation in PTI in*****A. thaliana***. (A, B) The kinetics of RbohD-dependent ROS production AtMPK3/6 activation. The vertical axis indicates relative values for the indicated event, and the horizontal axis the time after PAMPs elicitation. (C) RbohD-dependent ROS production and AtMPK3/6 activation are two signaling pathways in the early stage of PTI in *A. thaliana*. Both RbohD-dependent ROS production and AtMPK3/6 activation depend on BIK1. BIK1 directly regulates RbohD-dependent ROS production in PTI.

In response to flg22 or elf18, the kinetics of AtMPK3/AtMPK6 activation is similar to that of AtRBOHD-dependent ROS production (peaks within 5–10 min and diminishes after 30 min; as illustrated in Fig. 3 and references therein). However, accumulating evidence indicates that PAMP-activated AtMPK3/AtMPK6 may occur independently of the PAMP-triggered AtRbohD-dependent ROS production. Genomic screen and genome-wide gene expression profiling indicated that calcium-dependent protein kinases (CPKs) and MAPKs are probably activated independently downstream of the FLS2 receptor. CPKs, but not MPKs, may play a role in regulating ROS production in response to flg22 [139]. AtMPK3/AtMPK6 activation by flg22 (within 15 min) in seedlings of *atrbohD* mutants is comparable with wild-type [140]. Reciprocally, neither *atmpk3* nor *atmpk6* mutants show reduced flg22-triggered ROS production (0–40 min; [141]). Overexpression of the phosphatase AP2C1 could dephosphorylate and inactivate AtMPK3 and AtMPK6, but no alternation of ROS (0–48 min) was detected in two independent lines in response to flg22 [142]. Using conditional loss-of-function *atmpk3/atmpk6* double mutants, Xu et al., 2014 showed that the flg22-triggered ROS production (0–30 min) in wild-type plants and *atmpk3*/*atmpk6* double mutants is similar [143]. Furthermore, in *L-type lectin receptor kinase-VI.2* (*lecrk-VI.2–1*) mutant, activation of AtMPK3/AtMPK6 was reduced, but BIK1 phosphorylation and ROS production were not affected [144]. In *de-etiolated 3* (*det3*) mutants, flg22-induced ROS was reduced, whereas flg22-induced AtMPK3/AtMPK6 activation was enhanced [145]. In addition, results from *N. benthamiana* also showed that SIPK and WIPK are dispensable for ROS production in response to flg22 [146].

It seems that AtMPK3/AtMPK6 activation by elf18 is also dispensable for elf18-induced ROS production. Normal function of EFR requires endoplasmic reticulum (ER) quality control systems. Signaling defects in ER quality control of EFR biogenesis affect the full ROS production in response to elf18 [147], [148], [149], [150]. In *psl1-1* (encoding calreticulin3 in ER quality control signaling) mutants, elf18-induced ROS production (0–40 min) is retained at intermediate levels, whereas MAPK activation is much reduced [147], [150]. In *psl5–1* (encoding α-subunits of endoplasmic reticulum resident glucosidase II) mutants, elf18-induced ROS production (0–30 min) is strongly reduced below detectable levels, whereas an increase of MAPK activity is observed [148]. In the *Wassilewskija* (WS) ecotype *A. thaliana*, elf26-triggered ROS production (30 min) is not reduced in both *atmpk6* mutants and *atmkp1/atmpk6* (WS) double mutants [53]. Recently, it has been shown that in *anp2anp3* (WS) double mutants or β-estradiol-inducible *anp1anp2anp3* (WS) conditional triple mutants, elf18-induced production of ROS was compromised but activation of AtMPK3/AtMPK6 was normal [151]. Collectively, these data demonstrate that, in the early stage of PTI (about the first 30 min), PAMP-activated AtMPK3/AtMPK6 may occur independent of the PAMP-triggered ROS production.

In PTI, AtMPK4 can be activated by flg22, and it is assumed that AtMPK4 pathway positively regulates PTI [106]. The role of AtMPK4 in regulating ROS production in PTI is unclear. Experimental results suggest that flg22-induced ROS production was enhanced in *atmpk4* mutants [143], and *A. thaliana* expressing a constitutively active AtMPK4 showed a reduced ROS production in response to flg22 [152]. These results indicate that AtMPK4 negatively regulates ROS production in PTI. In another study, Kong et al. identified that the function of *AtMPK4* is dependent on *MEKK2*. ROS production induced by flg22 was comparable in wild type and *mekk2* mutants [107], indicating that disruption of AtMPK4-MEKK2 signaling pathway may not change AtRbohD-dependent ROS production in PTI. It is possible that mutations in the *MEKK2* gene and disinhibition of AtMEKK2 activation (in *atmpk4* mutants) can lead to different conclusions about the role of AtMPK4 in regulating ROS production in PTI.

## 6 Regulation of stomatal function by MAPK cascades in ROS signaling

**Fig. 4:**
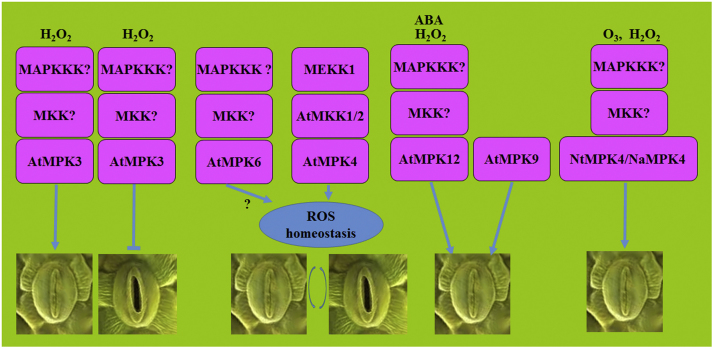
**Regulation of stomatal function by MAPK cascades and ROS signaling**. In H_2_O_2_ signaling, AtMPK3 functions both in inhibition of stomatal opening and in promotion of stomatal closure. Defective MEKK1-AtMKK1/AtMKK2-AtMPK4 cascade affects ROS homeostasis and affects stomatal function. AtMPK12, may act redundantly with AtMPK9, regulates ROS-mediated stomatal closure in response to ABA. NtMPK4 and NaMPK4 regulate stomatal closure in response to O_3_ and H_2_O_2_, respectively.

MAPK cascades have been shown to play a role in stomatal function in response to H_2_O_2_. AtMPK3 antisense plants are less sensitive to exogenous H_2_O_2_, both in inhibition of stomatal opening and in promotion of stomatal closure, suggesting a positive role of AtMPK3 in H_2_O_2_ signaling in stomata [153] (Fig. 4). AtMPK3 also regulates stomatal closure in response to *Pst* DC3000 [154] or *Xanthomonas campestris* [153], [155], likely acting downstream of H_2_O_2_ but independent of ABA. AtMPK6 is a positive regulator of flg22-triggered stomatal closure [152], although it is not clear if this function is related to H_2_O_2_. In the *atrbohD* mutant, flg22-induced AtMPK3 and AtMPK6 activation is not affected [106], [140], suggesting that AtMPK3 and AtMPK6 may not function downstream of AtRbohD-dependent ROS production in stomata. It has been shown that AtMPK6 is not involved in ABA-mediated stomatal closure [154]. In ABA inhibition of stomatal opening, AtMPK6 seems to participate in ABA-triggered H_2_O_2_ production downstream of AtMKK1 [71].

In *N. tabacum*, *NtMPK4*-silenced plants showed enhanced sensitivity to ozone with impaired regulation of stomatal closure [156]. In *N. attenuate*, silencing of *NaMPK4* gene impaired stomatal closure induced by exogenous H_2_O_2_ [157]. In seedlings of *A. thaliana*, *AtMPK4* is predominantly expressed in stomata [27], [158]. In immunity, AtMPK4 seems does not regulate stomatal closure in response to pathogens, as stomata in *AtMPK4*-overexpressing plants responded normally to *Pst* DC3000 or flg22 [152]. Loss-of-function of *MEKK1* resulted in H_2_O_2_ accumulation in stomata [62], [63], suggesting a role of MEKK1 in regulating ROS homeostasis in stomata (Fig. 4).

It is reported that the AtMPK9 and AtMPK12 act redundantly as positive regulators of ROS-mediated stomatal closure in response to ABA [159], yeast elicitor [160], or methyl jasmonate [161]. However, AtMPK9 and AtMPK12 may act via a different mechanism in stomata. It has been shown that AtMPK12 but not AtMPK9 can interact with MAPK phosphatase IBR5 [162]. Recent research indicates that AtMPK9 can be activated by autophosphorylation that may be independent of any upstream MAPKKKs or MKKs [163]. Furthermore, *AtMPK12* allele with an amino acid substitution alone could impair the ABA inhibition of opening [164].

## 7 Concluding remarks and perspectives

**Fig. 5:**
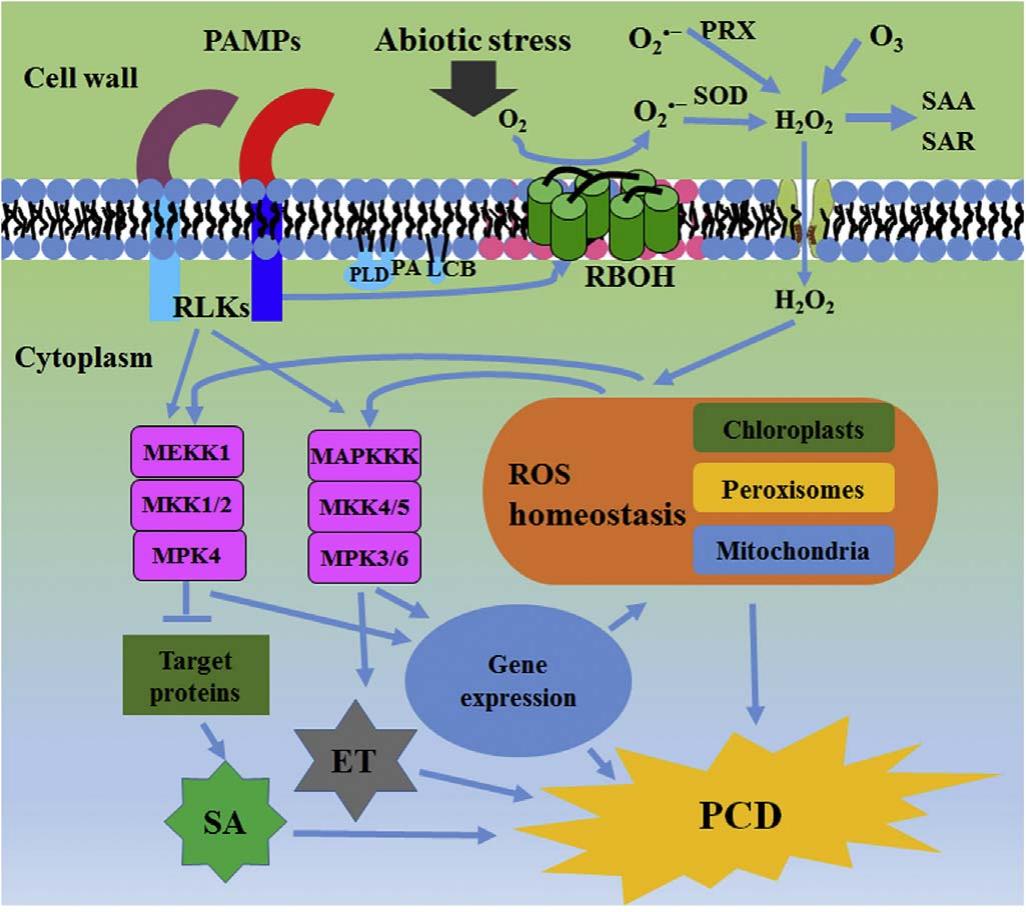
**Schematic representation of MAPK pathways in ROS signaling and responses**. In biotic or abiotic stresses, extracellular superoxide anion (O_2_^•−^) is induced by RBOH proteins. Superoxide anion (O_2_^•−^) can then dismutate to form hydrogen peroxide (H_2_O_2_) which acts as plant signaling component. Extracellular H_2_O_2_ can also be generated by PRX or O_3_·H_2_O_2_ may enter cells by aquaporins. Inside the cell, H_2_O_2_ triggers ROS signaling and responses (ROS-related gene expression and PCD) by disturbing ROS homeostasis. With different combinations of three tiers of kinases, timepoint-dependent activation, strength, and duration, MAPK signaling pathways act downstream of receptor-like kinases (RLKs) and ROS signaling to regulate ROS-related gene expression and PCD.

ROS production can be either harmful or beneficial to plants. Different types of ROS (H_2_O_2_, HO^•^, ^1^O_2_, or O_2_^•−^) and subcellular production sites (plastidic, cytosolic, peroxisomal, or apoplastic) may determine the physiological, biochemical, and molecular responses. The concentration of ROS and its interaction with other signaling components may also determine the specificity of ROS response. The complex role of MAPK cascades in ROS signaling and responses has been revealed in regulation of ROS-related genes, executing plant cell death, and modulating stomatal function. How ROS activates MAPK cascades still remains unclear. It is possible that plants not only use MAPK cascades to transduce ROS signaling to gene expression and sometimes cell death, but also initiate the negative feedback regulation by MAPK cascades to maintain ROS homeostasis. The different combinations of the three tiers of kinases, distribution, timepoint-dependent activation, strength, duration, and availability of substrates of MAPK cascades may determine the feed-forward or feed-back outcomes (Fig. 5).

Accumulating evidence indicates that, in *A. thaliana*, AtMPK3/AtMPK6 activation and AtRbohD-dependent ROS production are two independent pathways in the early stage of PTI (Fig. 3). Most recently, however, it was found that MKKK7, a MAPKKK, attenuates AtMPK6 activity and suppresses ROS production in response to flg22, through direct modulation of the FLS2 complex [165]. In response to wounding, AtMPK8 negatively regulates ROS production by repressing AtRbohD expression [84]. These results suggest a complicated relationship between MAPK signaling and AtRboh-dependent ROS production. Furthermore, both MAPK signaling and AtRboh-dependent ROS production have been implicated in O_3_[121]. Further studies are needed to unravel the whole picture about the role of MAPK signaling in Rboh-dependent apoplastic production of ROS.

A large body of research demonstrates that exogenous H_2_O_2_ can activate MAPK signaling (Table 1). It seems that exogenous application of H_2_O_2_ alone is not enough to induce the long-distance signaling manifested by AtRbohD-dependent ROS-triggered ROS waves [20], [22], [24]. Therefore, it is likely that activation of MAPK signaling by exogenous application of H_2_O_2_ is a relatively localized event. The molecular mechanism by which apoplastic ROS activate intracellular MAPK pathway needs further to be investigated.

Whereas ROS have been implicated in PCD in plant developmental processes and stress responses, the sub-cellular location and the mechanism of ROS generation during PCD are not clear. Compartment-specific ROS signals and communication between compartments may determine the outcome of ROS responses [11]. For instance, disturbance of the ROS homeostasis in chloroplasts, mitochondria, or peroxisomes tends to switch signaling to result in PCD [103], [104]. However, Rboh-dependent ROS appear to play a protection role in limiting the spread of PCD, e.g. in response to O_3_ or *Pst* DC3000 [121], [122]. It is speculated that low concentrations and restricted distribution of ROS mediates the role as signaling components to enhance cell survival and proliferation, whereas high concentrations of ROS (over a certain threshold) throughout the cell mediate the role as a death signal. MAPK cascades may act upstream of chloroplasts, mitochondria, or peroxisomes to regulate ROS homeostasis, or act downstream of these organelles to signal ROS response. In resistance to biotrophic or hemibiotrophic pathogens, MAPK cascades and ROS may elicit the escalated signaling at the interface of plant-pathogen interactions to induce HR that restricts pathogen access to water and nutrients. In response to necrotrophic pathogens or abiotic stresses, however, it is not yet known weather activation of MAPK cascades is simply to amplify the death signal. Furthermore, it remains to be seen how plants can discriminate between pathogens and beneficial microorganisms to initiate different MAPK activation and ROS responses.

PCD is a complex process that involves many signaling pathways. A strong interplay of ROS and MAPK cascades with other signaling molecules exists during plant PCD. It has been suggested that the HR is triggered only by balanced production of NO and ROS [166]. MAPK cascades are also involved in regulating NO signaling [87], [167]. ROS and SA act synergistically to drive HR [168], and the phenotype of spontaneous cell death in defect of MEKK-AtMKK1/2-AtMPK4 cascade result from accumulation of both ROS and SA [105], [158]. Ethylene signaling plays an important role in cell death that involves MAPK cascades [169], [170], [171]. Furthermore, crosstalk of ROS and MAPK cascades with calcium and lipid signaling fine-tunes the biological responses. The hierarchy of these events remains to be further elucidated. Although many questions still remain unanswered, further research will increase our understanding of the role of MAPK cascades in ROS signaling and responses.

## Conflict of interest

No conflicts of interest to declare.

## Figures and Tables

**Table 1:** Activation of MAPK signaling in response to ROS alteration or H_2_O_2_.

**Plant species**	**H**_**2**_**O**_**2**_**application or ROS alteration**	**Activation or responses of MAPK signaling**	**Reference (s)**
*Arabidopsis thaliana*	2 mM H_2_O_2_	MEKK1	Nakagami et al. [63]
*Arabidopsis thaliana*	200 μM H_2_O_2_	ANP1 (MAPKKK)	Kovtun et al. [38]
*Arabidopsis thaliana*	4 mM H_2_O_2_	AtMKK4-AtMPK6	Doczi et al. [39]
		AtMKK3-AtMPK7	
*Arabidopsis thaliana*	5 mM H_2_O_2_	AtMPK1/AtMPK2	Ortiz-Masia et al. [61]
*Arabidopsis thaliana*	200 μM, 1 mM, 2 mM, 4 mM, 10 mM, or 20 mM H_2_O_2_	AtMPK3/AtMPK6	Desikan et al. [60]; Doczi et al. [39]; Gupta and Luan [58]; Kim et al. [172]; Kovtun et al. [38]; Moon et al. [173]; Nakagami et al. [63]; Rentel et al. [40]; Wang et al. [167]; Yuasa et al. [174]
*Arabidopsis thaliana*	Ozone (250 nl L^−1^)	AtMPK3/AtMPK6	Ahlfors et al. [41]
*Arabidopsis thaliana*	Oxygen deprivation and reoxygenation	AtMPK3/AtMPK4/AtMPK6	Chang et al. [36]
*Arabidopsis thaliana*	20 mM H_2_O_2_	AtMPK4	Desikan et al. [60]
*Arabidopsis thaliana*	50 μM H_2_O_2_	AtMPK12	Jammes et al. [159]
*Arabidopsis thaliana*	1 mM H_2_O_2_	Inactivating AtPTP1	Gupta and Luan [58]
*Brassica napus*	5 mM H_2_O_2_	BnMPK4	Zhang et al. [64]
*Medicago sativa*	2 mM H_2_O_2_	OMTK1 (MAPKKK), MKK3 (MAPK)	Nakagami et al. [175]
*Nicotiana tabacum*	20 mM H_2_O_2_	46 kDa MAPK (SIPK)	Samuel et al. [42]
*Nicotiana tabacum*	Ozone (200 or 500 nl L^−1^)	46 kDa MAPK (SIPK)	Samuel et al. [42]
*Nicotiana tabacum*	10 mM GSH, 1 mM GSSG, or expressing StGCL-GS	WIPK/SIPK	Matern et al. [49]
*Oryza sativa*	10 mM H_2_O_2_	OsMPK1/OsMPK3/OsMPK6	Shi et al. [176]; Xie et al. [177]
*Pisum sativum*	5 mM H_2_O_2_	PsMPK2	Ortiz-Masia et al. [178]
*Solanum lycopersicon*	*SlRboh1*-VIGS	Inhibiting SlMPK1/SlMPK2 activation	Nie et al. [179]
*Zea mays*	10 mM H_2_O_2_	ZmMPK3/ZmMPK5	Lin et al. [91]; Wang et al. [180]; Zhang et al. [181]

*GSH* Reduced glutathione; *GSSG* Oxidized glutathione; *StGCL-GS* Bifunctional enzyme for glutathione synthesis in *Streptococcus thermophilus*; *VIGS* Virus-induced gene silencing.

**Table 2:** Alteration of MAPK signaling affecting ROS responses.

**Plant species**	**Alteration of MAPK signaling**	**ROS responses**	**Reference (s)**
*Arabidopsis thaliana*	*anp2/anp3* mutant (WS)	Increasing O_2_^•−^ production; Inhibiting H_2_O_2_ production in response to OGs or elf18; Over-accumulating Fe-SOD1, Mn-SOD, DHAR1, and CPN20	Savatin et al. [151]; Takac et al. [182]
*Arabidopsis thaliana*	*anp1/anp2* or *anp1/anp3* mutant (WS); *anp1*/*anp2*/*anp3* (conditional) mutant	Inhibiting H_2_O_2_ production in response to OGs or elf18	Savatin et al. [151]
*Arabidopsis thaliana*	*atmkk1*	Inhibiting CAT1 expression and H_2_O_2_ accumulation in response to ABA, drought, and salt stresses	Xing et al. [70]
*Arabidopsis thaliana*	*AtMKK1*-or *AtMPK6*-overexpressing	Promoting CAT1 expression and H_2_O_2_ accumulation in response to ABA	Xing et al. [70], [71]
*Arabidopsis thaliana*	*AtMKK5*-overexpressing	Promoting *CSD1/CSD2* expression under high light stress	Xing et al. [14]
*Arabidopsis thaliana*	*AtMKK5*-RNAi	Hypersensitive to ozone; Inhibiting *CSD1/CSD2* expression under high light stress; Inhibiting salt-induced *FSD2/FSD3* expression	Miles et al. [183]; Xing et al. [14]; Xing et al. [15]
*Arabidopsis thaliana*	*atmkp1* mutant (WS)	Enhancing ROS production in response to elf26	Anderson et al. [53]
*Arabidopsis thaliana*	*atmkp2* mutant	Over-accumulating H_2_O_2_ or O_2_^•−^ in response to MV	Lumbreras et al. [56]
*Arabidopsis thaliana*	*AtMKP2*-RNAi	Hypersensitive to ozone	Lee and Ellis [55]
*Arabidopsis thaliana*	*AtMPK6*-RNAi	Hypersensitive to ozone; Over-accumulating antioxidant proteins in response to ozone	Miles et al. [184]; Miles et al. [119]
*Arabidopsis thaliana*	*atmpk3* mutant	Suppressing H_2_O_2_ accumulation in SIT1- overexpressing seedlings	Li et al. [185]
*Arabidopsis thaliana*	*atmpk6* mutant	Inhibiting H_2_O_2_-induced NO generation; Inhibiting CAT1 expression and H_2_O_2_ accumulation in response to ABA; Promoting primary root elongation in response to H_2_O_2_; Suppressing H_2_O_2_ accumulation in SIT1- overexpressing seedlings	Han et al. [186]; Li et al. [185]; Wang et al. [167]; Xing et al. [71]
*Arabidopsis thaliana*	*atmpk8* mutant	Over-expression of *AtRbohD* and *OXI1*, and over-accumulating H_2_O_2_ in response to wounding	Takahashi et al. [84]
*Arabidopsis thaliana*	*AtMPK8*-GFP-overexpressing	Decreasing H_2_O_2_ accumulation in response to wounding	Takahashi et al. [84]
*Arabidopsis thaliana*	*atmpk9/atmpk12* mutant	Blocking H_2_O_2_-induced stomatal closure	Jammes et al. [159]
*Arabidopsis thaliana*	DEX-inducible *AtMKP2*-RNAi	Hypersensitive to ozone	Lee and Ellis [55]
*Arabidopsis thaliana*	Guard cell-specific *AtMPK3-*RNAi	Inhibiting stomatal response to H_2_O_2_	Gudesblat et al. [153]
*Arabidopsis thaliana*	*mekk1*, *atmkk1/atmkk2*, or *atmpk4* mutant	Accumulating H_2_O_2_; Alteration of ROS -related genes	Bjornson et al. [73]; Gao et al. [105]; Gawronski et al. [69]; Ichimura et al. [62]; Nakagami et al. [63]; Pitzschke et al. [68]
*Arabidopsis thaliana*	*mkkk20* mutant	Over-accumulating H_2_O_2_ in response to salt stress	Kim et al. [172]
*Arabidopsis thaliana*	Overexpressing DEX-inducible NtMEK2^DD^, AtMKK4^DD^, or AtMKK5^DD^	Over-accumulating H_2_O_2_ and HR-like cell death	Ren et al. [98]
*Arabidopsis thaliana*	*MKKK7*^*DD*^- overexpressing	Inhibiting ROS production in response to flg22	Mithoe et al. [165]
*Arabidopsis thaliana*	*PD98059* treatment	Inhibiting G/GO-induced *gst1* and *pal1* expression	Grant et al. [18], [187]
*Glycine max*	*GmMPK4-*VIGS	Accumulating H_2_O_2_	Liu et al. [188]
*Nicotiana attenuata*	*NaMPK4*-RNAi	Inhibiting H_2_O_2_-induced stomatal closure	Hettenhausen et al. [157]
*Nicotiana benthamiana*	Transient expression of *NbMEK1*^*DD*^ or *StMEK2*^*DD*^	*NbRbohB* expression and NbRbohB-dependent ROS generation in response to INF1 or R3a/AVR3a	Adachi et al. [88]; Asai et al. [87]; Yoshioka et al. [86]
*Nicotiana benthamiana*	*SIPK-*VIGS; *NTF6-*VIGS	Reducing *NbRbohB* expression and NbRbohB-dependent ROS production in response to INF1	Asai et al. [87]
*Nicotiana benthamiana*	Transient expression of *BnMPK4*^*CA*^	Over-accumulating H_2_O_2_ and cell death	Zhang et al. [64]
*Nicotiana tabacum*	*NtMPK4*-RNAi	Hypersensitive to ozone	Gomi et al. [156]
*Nicotiana tabacum*	*OsIBR5*-overexpressing	Hypersensitive to H_2_O_2_ stress (100 mM)	Li et al. [189]
*Nicotiana tabacum*	Overexpressing DEX-inducible *NtMEK2*^*DD*^	Promoting chloroplastic H_2_O_2_ production under light and HR-like cell death	Liu et al. [103]
*Nicotiana tabacum*	*SIPK-*overexpressing	Reducing H_2_O_2_ accumulation in response to harpin	Samuel et al. [99]
*Nicotiana tabacum*	*SIPK*-overexpressing or *SIPK*-RNAi	Hypersensitive to ozone	Samuel and Ellis [118]
*Nicotiana tabacum*	*ZmMPK7*-overexpressing	Reducing H_2_O_2_ accumulation under osmotic stress	Zong et al. [190]
*Nicotiana tabacum*	*SIPK*-RNAi	Over-accumulating H_2_O_2_ in response to harpin	Samuel et al. [99]
*Solanum lycopersicon*	*SlMPK1*/*SlMPK2*-VIGS; *SlMPK2*-VIGS	Reducing H_2_O_2_ accumulation in response to BR	Nie et al. [179]
*Vicia faba*	SB203580 treatment	Blocking H_2_O_2_-induced stomatal closure	Jiang et al. [191]
*Zea mays*	PD98059 or U0126 treatment	Inhibiting BR-induced apoplastic H_2_O_2_ production	Zhang et al. [92]

BnMPK4^CA^: Constitutively active form of BnMPK4 protein; DEX: Dexamethasone; BR: Brassinosteroid; Elf18: Synthetic polypeptide that corresponds to the bacterial PAMP elongation factor-Tu; INF1: Elicitin produced by *Phytophthora infestans*; MV: Methyl viologen; NtMEK2^DD^: Constitutively active form of NtMEK2 protein; NO: nitric oxide; OGs: Oligogalacturonides; PD98059 or U0126: Inhibitor of MEK in mammalian cells; R3a/AVR3a: Potato R protein/*P. infestans* effector pair; RNAi: RNA interference; SB203580: Inhibitor of p38 (MAPK) in mammalian cells; VIGS: Virus-induced gene silencing; WS: Wassilewskija (WS) ecotype *A. thaliana*.
